# Entry Inhibition of Influenza Viruses with High Mannose Binding Lectin ESA-2 from the Red Alga *Eucheuma serra* through the Recognition of Viral Hemagglutinin

**DOI:** 10.3390/md13063454

**Published:** 2015-05-29

**Authors:** Yuichiro Sato, Kinjiro Morimoto, Takanori Kubo, Takemasa Sakaguchi, Akira Nishizono, Makoto Hirayama, Kanji Hori

**Affiliations:** 1 Faculty of Pharmacy, Yasuda Women’s University, 6-13-1 Yasuhigashi, Asaminami-Ku, Hiroshima 731-0153, Japan; E-Mails: sato-y@yasuda-u.ac.jp (Y.S.); mori-k@yasuda-u.ac.jp (K.M.); kubo-t@yasuda-u.ac.jp (T.K.); 2 Department of Virology, Institute of Biomedical & Health Sciences, Hiroshima University, 1-2-3 Kasumi, Minami-Ku, Hiroshima 734-8551, Japan; E-Mail: tsaka@hiroshima-u.ac.jp; 3 Department of Microbiology, Faculty of Medicine, Oita University, 1-1 Idaigaoka, Hasama-machi, Yufu, Oita 879-5593, Japan; E-Mail: a24zono@oita-u.ac.jp; 4 Graduate School of Biosphere Science, Hiroshima University, 1-4-4 Kagamiyama, Higashi-Hiroshima 739-8528, Japan; E-Mail: hirayama@hiroshima-u.ac.jp

**Keywords:** lectin, red algae, *Eucheuma serra*, high mannose glycan, anti-influenza activity

## Abstract

Lectin sensitivity of the recent pandemic influenza A virus (H1N1-2009) was screened for 12 lectins with various carbohydrate specificity by a neutral red dye uptake assay with MDCK cells. Among them, a high mannose (HM)-binding anti-HIV lectin, ESA-2 from the red alga *Eucheuma serra*, showed the highest inhibition against infection with an EC_50_ of 12.4 nM. Moreover, ESA-2 exhibited a wide range of antiviral spectrum against various influenza strains with EC_50_s of pico molar to low nanomolar levels. Besides ESA-2, HM-binding plant lectin ConA, fucose-binding lectins such as fungal AOL from *Aspergillus oryzae* and AAL from *Aleuria aurantia* were active against H1N1-2009, but the potency of inhibition was of less magnitude compared with ESA-2. Direct interaction between ESA-2 and a viral envelope glycoprotein, hemagglutinin (HA), was demonstrated by ELISA assay. This interaction was effectively suppressed by glycoproteins bearing HM-glycans, indicating that ESA-2 binds to the HA of influenza virus through HM-glycans. Upon treatment with ESA-2, no viral antigens were detected in the host cells, indicating that ESA-2 inhibited the initial steps of virus entry into the cells. ESA-2 would thus be useful as a novel microbicide to prevent penetration of viruses such as HIV and influenza viruses to the host cells.

## 1. Introduction

Influenza viruses cause annual epidemics and occasional global pandemics. The viral envelope glycoprotein HA (hemagglutinin) functions to bind to cellular receptors and mediate fusion with endosomal membranes [1]. Regarding many viruses that are of public health concern, viral envelope proteins are glycosylated with high mannose (HM) glycans. For instance, HIV envelope gp120, which mediates the direct binding to the CD4 receptor, is highly glycosylated with HM glycans [2]. High levels of HM glycans are present on viral surface glycoproteins of both HCV and SARS-CoV [3,4]. For influenza virus, HM glycans are not abundant on viral HA but they are reportedly present near the receptor binding region of the HA1 subunit [5]. Accordingly, HM glycans on viral surface are attractive targets for carbohydrate-based antiviral reagents [6].

The recently discovered HM-binding lectin family from lower organisms such as bacteria, cyanobacteria, and marine algae represents a novel class of antiviral compounds. This family includes cyanobacterial lectin: OAA from *Oscillatoria agardhii* [7,8,9,10] and its homologous proteins such as bacterial lectin: PFL from *Pseudomonas fluorescens* Pf0-1 [11] and BOA from *Burkholderia oklahomensis* [12], red algal lectins: ESA-2 from *Eucheuma serra* [13] and KAA-2 from *Kappaphycus alvarezii* [14]. They have commonly two or four tandem repeats consisting of highly conserved sequences but lack homology to any other existing protein families. This family of proteins shows a unique β-barrel-like topology [8]. Some of these lectins including OAA and ESA-2 have been reported to exhibit strong anti-HIV activity by inhibiting the initial step of virus entry into the host cells with EC_50_s of low nanomolar range by directly binding to gp120 [7,13]. Structural insights of lectin-carbohydrate interaction for this family and the molecular basis of anti-HIV properties have been investigated. For example, х-ray structure of the ligand-bound form of anti-HIV lectin BOA has revealed that hydrogen bonds are associated with the core trisaccharide comprising Manα(1–3)Manα(1–6)Man, which is the part of the D2 arm of Man-9 [12]. Carbohydrate binding specificity of this protein family has been evaluated by centrifugal ultrafiltration method using fluorescent-labeled oligosaccharides in the aqueous phase or glycan array analysis in the solid phase [7,11,13,14]. Both analyses support the observation in X-ray structure that the trimannosyl unit is a primary recognition center. Additionally, α1–2Man at the non-reducing terminal of this trisaccharide disrupts the interaction between lectin and oligosaccharide. This lectin family is thus independent of other known HM-binding anti-HIV lectins such as cyanovirin-N (CV-N) from *Nostoc ellipsosporum* or GRFT from *Griffithsia* sp. in terms of molecular structure and carbohydrate specificity. CV-N, the most extensively characterized cyanobacterial lectin, recognizes Manα1–2Man termini of HM-glycans, and shows a wide range of antiviral activity against HIV, Ebola, HCV as well as influenza viruses [15,16,17,18]. Red algal GRFT, which binds to even monosaccharides as well as HM-glycans, displays broad spectrum HIV-1 inhibitory activity without altering gene expression and cytokine production [19].

Besides strong anti-HIV activity found in OAA lectin family, potent anti-influenza virus activity has also been demonstrated in PFL and KAA-2 [11,14]. However, red algal lectin ESA-2 has not been studied for its inhibitory effect of influenza virus infection. In the present study, we have examined the anti-influenza potency of ESA-2 by comparing those of various lectins with diverse carbohydrate-binding specificity. Furthermore, the molecular basis of anti-influenza activity of ESA-2 was also evaluated from the aspects of lectin-envelope glycoprotein interactions.

## 2. Results and Discussion

### 2.1. Anti-Influenza Activity of Various Lectins with Diverse Carbohydrate Specificity

**Figure 1:**
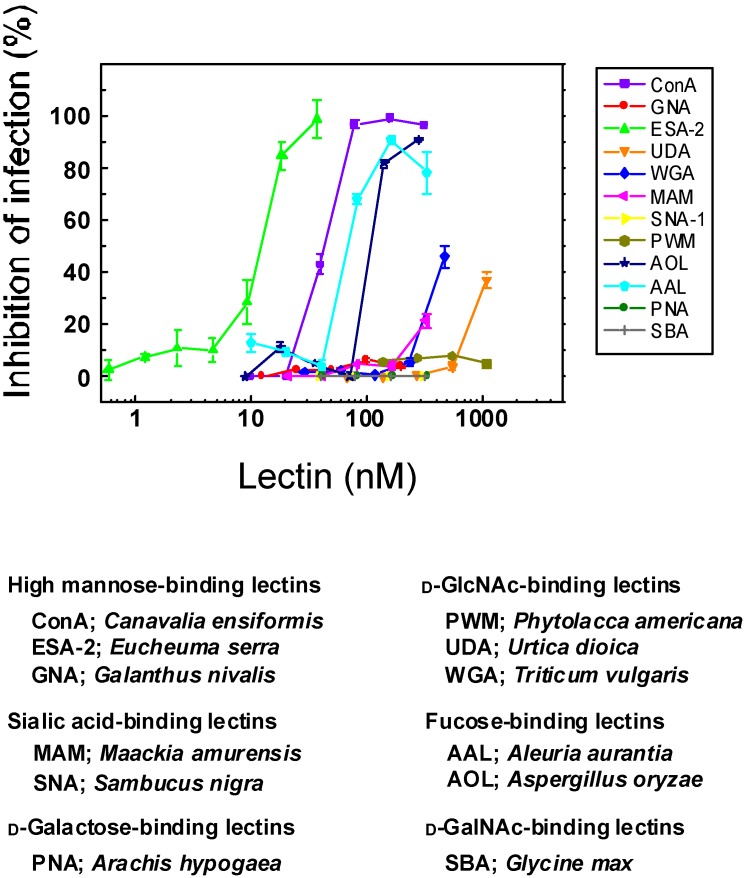
Anti-influenza activity of various lectins with diverse carbohydrate specificity. Madin-Darby canine kidney (MDCK) cells were infected with A/Oita/OU1 P3-3/09 (H1N1-2009) in the presence of various concentrations of lectins. Dose dependent effect of lectins on the viability of virus-infected cells was determined by neutral red (NR) dye uptake assay. Mock-infected cells were used as controls. EC_50_ values of ESA-2, ConA and AAL were 12.4 nM, 40.4 nM and 67.0 nM, respectively, and the means were significantly different (*p* < 0.05) as revealed by one way ANOVA analysis using the software (Origin. ver. 6.0).

In 2009, a novel influenza virus of H1N1 subtypes emerged and caused pandemics all over the world [1]. Lectin sensitivity profile of this swine origin H1N1 influenza virus (H1N1 2009) was examined by utilizing twelve lectins with various carbohydrate-binding specificities. In this test, we employed neutral red (NR) dye uptake assay using the clinical isolates of H1N1-2009 virus, A/Oita/OU1 P3-3/09. Among the lectins tested, high mannose (HM)-binding red algal lectin, ESA-2 from *Eucheuma serra* showed the highest potency to inhibit influenza virus infection, showing an EC_50_ of 12.4 nM (Figure 1). This lectin specifically recognizes branched structure of HM *N*-glycans including trisaccharide comprising Manα(1–3)Manα(1–6)Man in the D2 arm as a primary target [13]. ESA-2 is devoid of monosaccharide binding including mannose, as well as other *N*-glycans such as complex type or hybrid type [13]. It is therefore likely that certain HM glycan(s) present on the region of virus surfaces that are involved in receptor binding or on the critical position for virus infection would be a specific target of ESA-2. In fact, site specific occurrence of HM glycans on HA1 subunit has been reported [5]. We have previously demonstrated that ESA-2 has four binding sites in its molecule that are built from four tandem repeats with characteristic homologous sequence in this lectin family [13]. Another bacterial lectin in this family, PFL from *Pseudomonas fluorescens* Pf0-1, which has only two carbohydrate binding sites but with the same carbohydrate specificity as ESA-2, has been shown to exhibit less anti-influenza activity compared with ESA-2 [11]. This implies that higher valency of lectins would be important for effective capturing of virus particles. Similarly, an obligate dimeric construct of CV-N shows enhanced inhibition of HIV-1 fusion compared with wild type CV-N monomer [20]. Likewise, oligomeric states of GRFT affect the HIV inhibitory potency of GRFT in which a monomeric variant of GRFT failed to inhibit HIV infection [21,22].

As shown in Figure 1, d-GlcNAc-binding lectins including UDA from *Urtica dioica* and WGA from *Triticum vulgaris* exhibited very weak antiviral activity with one or two order less magnitude in comparison with ESA-2. Human influenza viruses preferentially recognize α2.6 linked sialic acid moiety (SAα 2.6Gal) on epithelial cells in the human trachea whereas avian influenza viruses prefer SAα2,3Gal moiety, that are present on the cells in the intestinal tract of waterfowls [23,24,25]. One of the sialic acid-binding lectins, MAM from *Maackia amurensis*, which preferentially recognizes α2.3 linkage, showed very weak activity, possibly due to the blockade of cell surface receptor, rather than the direct binding to the virus particles. In contrast, α2.6 linked sialic acid-binding lectin SNA from *Sambucus nigra* failed to protect cells from virus infection. Inhibition profiles of sialic acid-binding lectins against this H1N1-2009 strain remain controversial but both of them were deemed as virtually inactive for the inhibition of influenza infection. HM-binding legume lectin ConA showed a moderate antiviral activity with EC_50_ of 41.3 nM. Fucose-binding lectins such as AOL from *Aspergillus oryzae* and AAL from *Aleuria aurantia* were also inhibitory with EC_50_s of 50–100 nM but the inhibitory activity of fucose-binding lectins was much weaker compared with ESA-2. It has been reported that site-specific glycosylation of the HA by mammalian cells are dependent on various factors such as amino acid sequence of HA, accessibility of the glycosyl transferases, and the conformational position of glycosylation site in the HA structure [5]. The widespread occurrence of reducing terminal fucose residue(s) at a variety of glycosylation sites in HA1 subunit may account for the antiviral activity of fucose-binding lectins. It has been reported that some influenza viruses bound to fucose-branched SAα2.3Gal glycan receptors much stronger than linear SAα2.3Gal glycans [26]. Therefore, it might be also possible that fucose-binding lectin exerts its anti-influenza activity by blocking fucose-containing cell receptors.

### 2.2. Anti-Influenza Activity of Red Algal Lectin ESA-2

**Figure 2:**
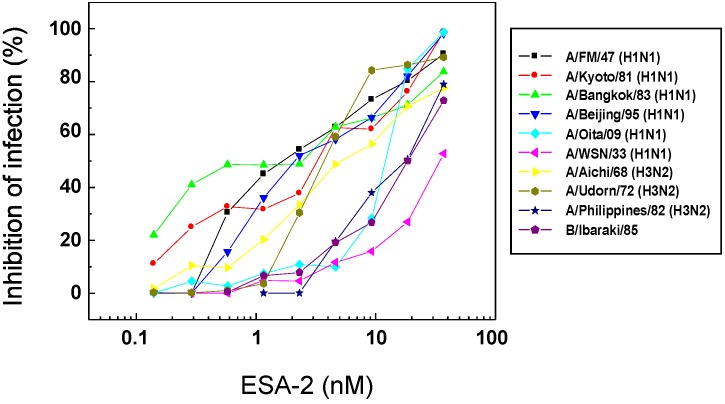
Profiles of anti-influenza activity of ESA-2 against various influenza virus strains. Effect of ESA-2 on cell viability after 48 h post infection of various influenza viruses was determined using the NR dye uptake assay. Percent inhibition of infection is calculated as the average value of duplicate assays.

To explore further antiviral activity of ESA-2, NR dye uptake assay was performed with various influenza virus strains. As shown in Figure 2, ESA-2 strongly inhibited infection caused by all of the influenza virus strains except for an earlier laboratory-adapted strain, A/PR8/34 (H1N1). The lack of CV-N binding to the HA1 and resistance to CV-N observed in the A/PR8/34 strain have been accounted for by the absence of HM glycans near the cellular binding region of HA1 [18]. The antiviral profile of ESA-2 was similar to that of the red algal lectin, KAA-2 from *Kappaphycus alvarezii* which belongs to the same anti-HIV lectin family [14]. ESA-2 showed a broad spectrum of anti-influenza virus activity with EC_50_s of pico—to low nano-molar range (Table 1). Cytotoxicity of ESA-2 was not observed up to 1000 nM, the highest dose in this experiment. In contrast, antiviral activity of fucose-binding AOL varied significantly depending on the virus strains (Table 1). Besides A/PR8/34, A/Udorn/72 and B/Ibaraki/2/85 were insensitive to AOL. In contrast, A/Philippines/2/82 and A/WSN/33 were highly susceptible to AOL.

**Table 1:** *In vitro* activity of ESA-2 and AOL against various influenza strains.

Virus	Strain	ESA-2	AOL
		EC_50_ (nM)	EC_50_ (nM)
**Influenza A**	PR8/34 (H1N1)	−^a^	−
	FM/1/47 (H1N1)	0.8 ± 0.2	202.9 ± 26.8
	Kyoto/1/81 (H1N1)	2.7 ± 0.9	196.8 ± 26.6
	Bangkok/10/83 (H1N1)	2.8 ± 1.5	78.4 ± 23.6
	Beijing/262/95 (H1N1)	1.7 ± 0.4	19.2 ± 10.5
	Oita/OU1 P3-3/09 (H1N1)	12.4 ± 0.4	123.1 ± 25.4
	WSN/33 (H1N1)	34.6 ± 2.7	1.5 ± 3.2
	Aichi/2/68 (H3N2)	5.2 ± 1.5	200.8 ± 25.7
	Udorn/72 (H3N2)	3.7 ± 0.4	−
	Philippines/2/82 (H3N2)	17.2 ± 3.9	1.1 ± 2.5
**Influenza B**	Ibaraki/2/85	20.4 ± 3.3	−

^a^ PR8/34 was insensitive to ESA-2 and AOL up to 75 nM and 285.7 nM, the highest doses in this experiment, respectively.

### 2.3. Evaluation of ESA-2 Potency as an Entry Inhibitor for Influenza Virus

**Figure 3:**
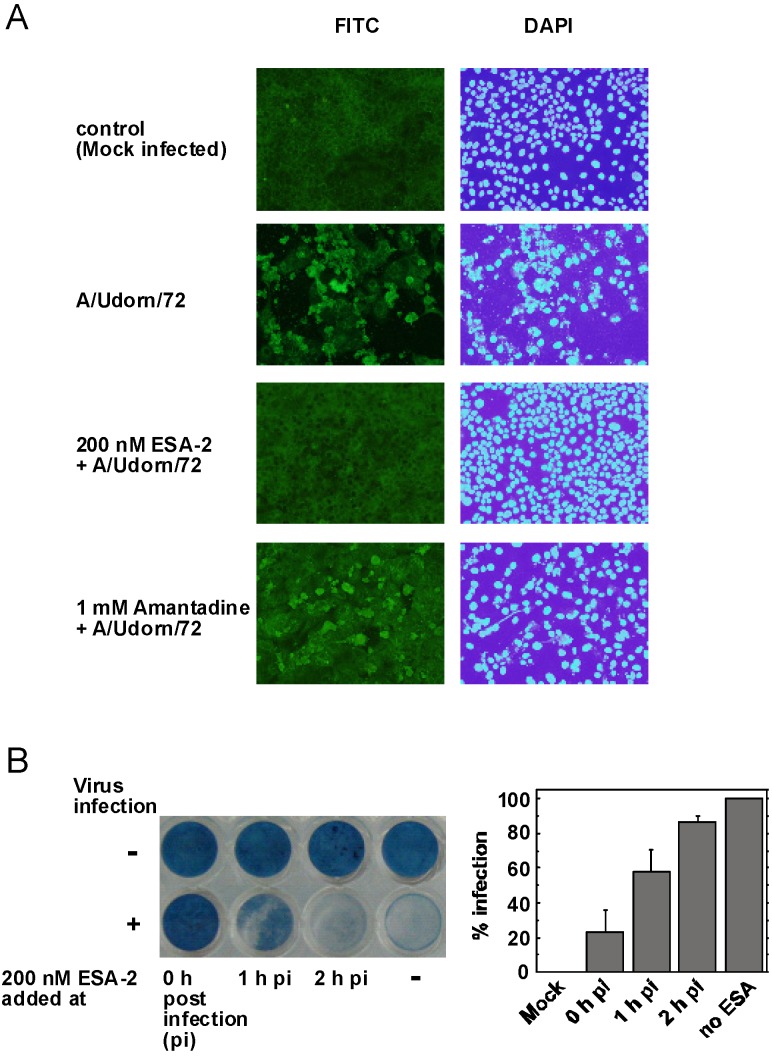
(**A**) Inhibition of influenza virus invasion into MDCK cells by ESA-2. MDCK cells grown on cover slips were infected with A/Udorn/72(H3N2) in the presence of 200 nM ESA-2 or 1 mM amantadine. After 24 h post infection, the cells were fixed and the viral antigens in the cells were visualized with anti-hemagglutinin (HA) mouse monoclonal antibody followed by fluorescein isothiocyanate (FITC)-conjugated goat anti-mouse IgG. Nuclei within the cells were stained with DAPI (200× magnification); (**B**) Effect of ESA-2 addition after post-infection. H292 cells were infected with A/Udorn/72(H3N2) and after 1 or 2 h post-infection, 200 nM ESA-2 was added to the cells. Cell viability was evaluated by amide black staining followed by quantitation by image processing.

To examine whether ESA-2 inhibits virus entry into the cells, cellular distribution of viral antigen either in the presence or absence of ESA-2 was observed by immunofluorescence microscopy. In the presence of 200 nM ESA-2, no viral antigens were detected in the MDCK cells infected with A/Udorn/72 (Figure 3A). In addition, no cytopathic effect (CPE) in the infected cells was observed. In contrast, 1 mM amantadine, which is an M2 channel blocker but not an entry inhibitor of influenza virus, failed to block the viral infection in the host cells. To test whether ESA-2 inhibits initial virus entry but not another early step of viral replication, effect of ESA-2 was determined after post-infection. The data in Figure 3B clearly showed that ESA-2 failed to inhibit infection when it was administered to the cells after 1 or 2 h post-infection. In contrast, ESA-2 effectively inhibited virus replication when it was added to the cells simultaneously with the virus. These results indicate ESA-2 acts as an entry inhibitor.

### 2.4. ESA-2 Binding Studies with Viral Hemagglutinin (HA)

**Figure 4:**
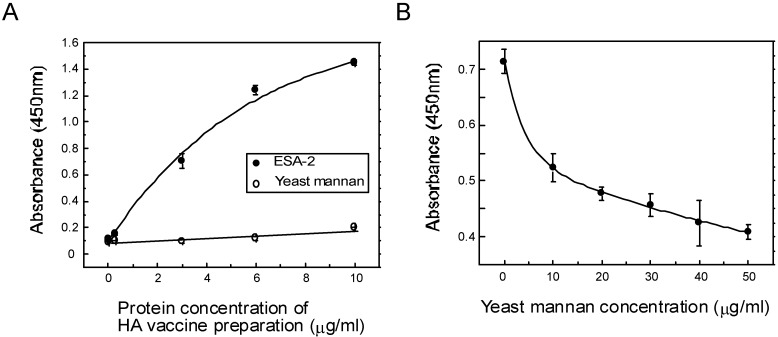
Interaction between ESA-2 and influenza virus glycoprotein HA. (**A**) Binding interaction between ESA-2 and HA was analyzed by an ELISA assay. Various dilutions of influenza vaccine preparation which contain HA mixture of A/California/7/09 (H1N1), A/Victoria/210/09 (H3N2), and B/Brisbane/60/08 was added to ESA-2 immobilized plate. Interactions were detected by incubating with mouse anti-HA monoclonal antibody followed by incubation with HRP-conjugated goat anti-mouse IgG antibody. The colorimetric substrate (TMB) was added to each well and the absorbance at 450 nm was measured. Yeast mannan (YM) was used as a reference; (**B**) Inhibition assay was performed using YM with aforementioned methods, except that the ESA-2 coated plate was pre-incubated with various concentrations of YM for 1 h at room temperature before adding vaccine preparation containing HA mixture.

**Figure 5:**
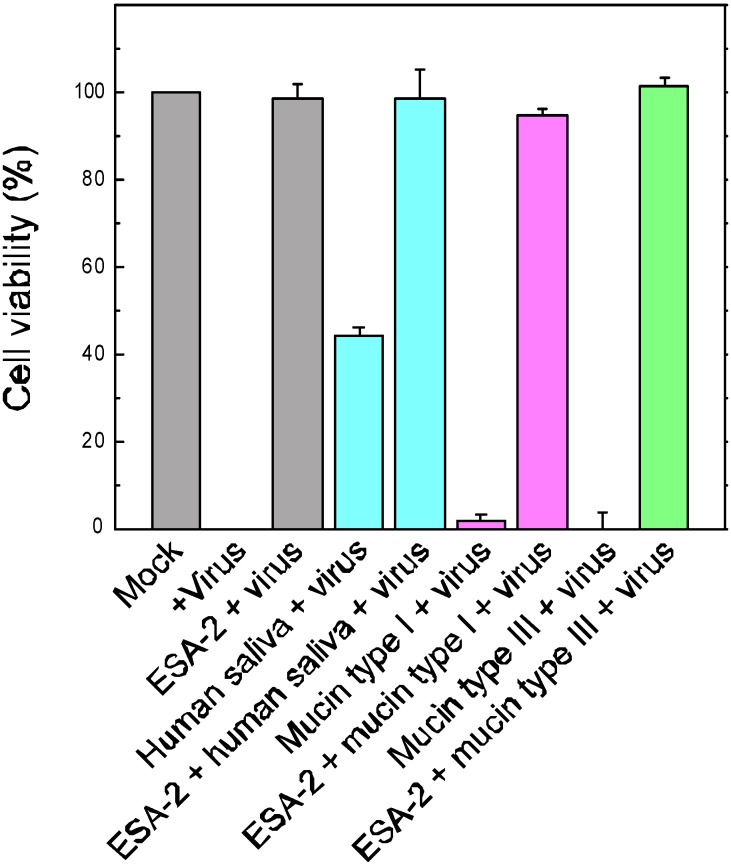
Effect of ESA-2 on viral infection in the presence of mucins or human saliva. H292 cells were infected with A/Udorn/72 for 24 h in the presence or absence of 200 nM ESA-2 in the DMEM containing 10 µg/mL of mucin type I (bovine submaxillary gland), mucin type III (porcine stomach), or human saliva (10× dilution). Cell viability was evaluated by amide black staining followed by quantitation by image processing. The data was shown as percentage of cell viability (mock infection; 100%, virus infection; 0%).

Direct interaction between ESA-2 and influenza envelope HA glycoproteins was demonstrated in ELISA assay (Figure 4). HA was dose-dependently bound to immobilized ESA-2 on the plate but not to the reference glycoprotein. This interaction was competitively inhibited by yeast mannan, a selective inhibitor of ESA-2, indicating ESA-2 bound to the HA through HM glycans. It has been demonstrated that CV-N exhibited anti-influenza activity by directly binding to HA1 [18]. Anti-influenza activity of ESA-2 exemplified in this study seems to be the same mechanism as that of CV-N. However, despite partially overlapping carbohydrate specificity for HM-glycans, CV-N and other HM-binding lectins, MVL from *Microcystis viridis* and GNA from *Galanthus nivalis* inhibited HCV infection through a different and complex mode of action [27]. In this respect, interaction of ESA-2 with cellular proteins, which potentially leads to various cellular responses such as proliferation or inflammation, should be taken into account in undertaking *in vivo* application. To evaluate the effectiveness of ESA-2 under conditions that model natural environment, we performed *in vitro* infection assays in the presence of mucins or human saliva. In this test, H292 cells were infected with A/Udorn/72 for 24 h and the cell viability was determined. As shown in Figure 5, 200 nM ESA-2 efficiently inhibited viral infection in the presence of mucin type I (bovine submaxillary gland), mucin type III (porcine stomach), and human saliva, although the saliva by itself inhibited the viral infection moderately.

This study provided the first evidence that the red algal lectin ESA-2 potently inhibited influenza virus propagation by directly binding to HM glycans on the envelope glycoprotein HA. Therefore, ESA-2 would be useful as a novel entry inhibitor for various viruses with HM glycans to prevent their transmission effectively. We assume that ESA-2 would be useful as a disinfectant or prophylactic agent (e.g., application as a spray reagent for the mask, *etc.*). We have to precisely investigate the safety and effectiveness of ESA-2 if ESA-2 could be developed as a topical microbicide to prevent virus infections. Future work will be directed to show such effectivity of ESA-2 using some animal models.

## 3. Experimental Section

### 3.1. Materials

The following lectins were purchased from Seikagaku Corporation (Tokyo, Japan). AAL: Aleuria aurantia, ConA: Canavalia ensiformis, MAM: Maackia amurensis, PNA: Arachis hypogaea, PWM: Phytolacca americana, SBA: Glycine max, WGA: Triticum vulgaris. The following lectins were purchased from Cosmo Bio (Tokyo, Japan). GNA: Galanthus nivalis, SNA: Sambucus nigra, UDA: Urtica dioica. The lectin AOL from Aspergillus oryzae was obtained from Tokyo Chemical Industry (Tokyo, Japan). ESA-2 from Eucheuma serra was prepared as described previously [13] and kept at 20 °C until used. Influenza virus vaccine for a mixture of A/California/7/09 (H1N1), A/Victoria/210/09 (H3N2), and B/Brisbane/60/08 was purchased from Denka-Seiken (Tokyo, Japan).

### 3.2. Cells and Viruses

The following viruses that had been stocked by one of the authors were examined: A/WSN/33 (H1N1), A/PR8/34 (H1N1), A/FM/1/47 (H1N1), A/Kyoto/1/81 (H1N1), A/Bangkok/10/83 (H1N1), A/Beijing/262/95 (H1N1), A/Aichi/2/68 (H3N2), A/Udorn/72 (H3N2), A/Philippines/2/82 (H3N2), and B/Ibaraki/2/85. A clinical isolate of the recently emerged H1N1 strain, A/Oita/OU1 P3-3/09, was also examined. All viruses have been propagated in embryonated eggs. The aliquots of each virus preparation were used for the assay. Madin-Darby canine kidney (MDCK) cells or human lung carcinoma NCI-H292 (H292) cells were grown as host cells for infection studies in Dulbecco’s modified Eagle medium (DMEM) supplemented with 10% fetal bovine serum and penicillin-streptomycin.

### 3.3. Determination of Anti-Influenza Activity of Various Lectins

Anti-influenza activities of lectins were determined by the neutral red (NR) dye uptake assay as described previously [14]. Various concentrations of lectins were prepared with DMEM containing 10 µg/mL trypsin and added to MDCK cells cultured in a 96-well microplate. Subsequently, influenza virus (A/Oita/OU1 P3-3/09) was inoculated to each well at a multiplicity of infection of approximately 0.001. The cells were incubated at 37 °C for 48 h and NR dye was added to be incorporated into the survived cells. After incubating the cells with 1% formaldehyde containing 1% CaCl_2_, the incorporated dye was eluted from the cells with 1% acetic acid/50% ethanol and the color intensity for each well was measured at 540 nm with a microplate reader (1420 multilabel counter, PerkinElmer, Waltham, MA, USA).

Alternatively, the infected H292 cells were fixed with acetone and stained with amide black solution (0.5% amide black, 45% ethanol, 10% acetic acid). The stained cells in each well were pictured with greyscale, and the color intensity was quantitated by image processing program NIH image.

To test the inhibitory profiles of ESA-2, NR dye uptake assay was performed as described above using nine influenza A virus strains and one influenza B virus strain. For comparison, the anti-influenza virus profile of fucose-binding lectin AOL was also evaluated in the same way.

*In vitro* infection assays in the presence of mucins or human saliva were peformed as follows. H292 cells were infected with A/Udorn/72 for 24 h in the presence of 200 nM ESA-2 in the DMEM containing 10 µg/mL of mucin type I (bovine submaxillary gland), 10 µg/mL of mucin type III (porcine stomach), or human saliva (10× dilution). Cell viability was evaluated by amide black staining as described above.

### 3.4. Evaluation of ESA-2 Potency as an Entry Inhibitor for Influenza Virus

To assess whether ESA-2 inhibited the initial steps of viral infection, immunofluorescence staining was performed to visualize the virus antigens in the host cells. Briefly, MDCK cells grown on cover slips were infected with A/Udorn/72(H3N2) at a multiplicity of infection of approximately 0.001 in the presence of 200 nM ESA-2 in DMEM containing 10 µg/mL trypsin. The cells were fixed with 80% acetone and the virus antigen was detected by incubating with mouse monoclonal anti-HA antibody (HyTest, Turku, Finland) followed by with fluorescein isothiocyanate (FITC)-conjugated goat anti-mouse IgG antibody (Anticorps Secondaires, Compiègne, France). The cells were mounted with Vectashield with 4,6-diamidino-2-phenylindole (DAPI) (Vector Laboratories, Burlingame, CA, USA) and were observed under a fluorescence-microscope (OLYMPUS BX51, Olympus, Tokyo, Japan).

### 3.5. ESA-2 Binding Studies with Viral Hemagglutinin (HA)

Enzyme-linked immunosorbent assay (ELISA) was performed to examine direct interaction of ESA-2 and HA of influenza virus. ESA-2 was immobilized on ELISA plates (BD Biosciences, Bedford, MA, USA) as described previously [14]. The wells were blocked with 3% skim milk and subsequently incubated with influenza vaccine preparation (Astellas, Tokyo, Japan), which contained HAs from three different influenza subtypes. The wells were incubated with mouse anti-HA monoclonal antibody (HyTest) followed by incubation with horse-radish peroxidase (HRP)-conjugated goat anti-mouse IgG antibody (GE Healthcare, Buckinghamshire, UK). To detect the interaction, a TMB substrate for HRP, 3,3,5,5-tetramethylbenzidine (Sigma-Aldrich, Saint-Louis, MO, USA), was added to each well and absorbance at 450 nm was measured using a microplate reader (1420 multilabel counter). For the inhibition study, mannan from yeast was added to the ESA-2 coated plate prior to incubation with HA vaccine preparation.

## 4. Conclusions

In conclusion, the red algal lectin ESA-2 from *E. serra* exerts its anti-influenza activity by strongly inhibiting cell entry of various influenza viruses in a strain independent manner by directly binding to HM glycans on the envelope glycoprotein HA. Given that ESA-2 exhibits a broad spectrum of anti-influenza activity as well as potent anti-HIV activity, this protein would be a promising candidate as an entry inhibitor for prophylactic antiviral therapy.
